# Long-Distance Transport of Prosystemin Messenger RNA in Tomato

**DOI:** 10.3389/fpls.2017.01894

**Published:** 2017-11-06

**Authors:** Haiyan Zhang, Yuanyuan Hu

**Affiliations:** ^1^Tianjin Key Laboratory of Animal and Plant Resistance, College of Life Sciences, Tianjin Normal University, Tianjin, China; ^2^College of Life Science, Shanxi Normal University, Linfen, China

**Keywords:** prosystemin, systemin, long-distance transport, wounding response, agro-infiltration

## Abstract

*Main conclusion:* The transcripts of transgenic *prosystemin* (*PS*) gene are mobile and the *PS* mRNA can be translated into protein in tomato and tobacco plants. Systemin (SYS) and its precursor protein, prosystemin (PS), are upstream components of the wound-induced signaling pathway in tomato. Although the mobile signal(s) for wound responses has been the subject of considerable research, its identity remains controversial. Intensive studies have revealed the essential role of mRNA on plant systemic signaling. We hypothesize that *PS* mRNA can act as a transmissible signal in tomato. Herein, we demonstrated that transgenic *PS* mRNA occurs in leaves located at considerable distances from the initial site of its generation by a transient *Agrobacterium*-infiltration assay system. We also showed that PS protein is present in the vascular bundle of the distant leaves. Our results indicate that transgenic *PS* mRNA may be functional as a long-distance signal to modulate systemic defense responses in tomato, providing novel insights into the multifaceted systems by which SYS signaling transports.

## Introduction

Plants respond to mechanical wounding, herbivore injury or pathogen infection by induction of multiple protective genes throughout the whole plant as a defense against the invasion. This phenomenon implies the existence of complex regulatory networks that are capable of generating, transporting, and interpreting alarm signals produced at the plant–herbivore/pathogen interface. Systemic defense responses thus provide an attractive model system for studies of cell-to-cell signal transduction pathways that operate over long distances (Li et al., 2002).

Systemin (SYS), an octadeca-peptide isolated from tomato, was the first bioactive peptide hormone identified in plants (Pearce et al., 1991). The 18-amino acid SYS is thought to be processed from a 200-amino acid precursor protein called prosystemin (PS) by proteolytic cleavage upon wounding of tomato leaves (McGurl et al., 1992). Perception of SYS by its membrane receptor triggers a complex cascade of intracellular events that are all orchestrated to activate a phospholipase A2 (PLA_2_) for the release of linolenic acid (LA) from plasma membrane. LA is subsequently converted to oxylipins 12-oxy-phytodienoic acid (OPDA) and jasmonic acid (JA) that regulate the transcription of defense-related genes (Ryan, 2000). Previous studies showed that application of SYS enhances JA biosynthesis and in turn, JA treatment increases abundance of PS protein (Howe et al., 1996). In addition, SYS coordinates synthesis of many other immunoregulatory signals such as ethylene (ET), hydrogen peroxide, cytosolic calcium ion influx, and plasma membrane depolarization (Ryan, 2000; Huffaker, 2015).

Prosystemin (*PS*) gene expression occurs tissue-specifically in vascular bundles and unwounded tomato plants exhibit constitutive expression of *PS* at a low level (McGurl et al., 1992). The tomato plants transformed with a *PS* antisense gene, driven by the constitutive *35S* promoter, exhibited a sharply compromised wound response that leads a reduced defense against *Manduca sexta* larvae attack (McGurl et al., 1992). On the other hand, plants transformed with *PS* cDNA in its sense orientation, produced high levels of *PS* mRNA in plants (McGurl et al., 1994), resulting in constitutively expressing of more than 20 systemic wound response proteins in leaves as if they are in a permanently wounded state. Wild-type (WT) plants grafted as scions onto transgenic rootstalks that constitutively express the *PS* sense gene also accumulate high levels of defense proteins in the absence of wounding, suggesting that a systemic signal is produced by the transgenic rootstocks and is transported to the WT scions (McGurl et al., 1994).

SYS was originally known as the primary long-distance transmissible signal in plants as a synthetic ^14^C-labeled SYS exogenously supplied to wound sites was shown to be mobile, traveling systemically from a wounded leaf to the upper leaves of treated tomato plants (Pearce et al., 1991). This is compatible with results obtained in poplar trees where the systemic wound signal also travels through the phloem to activate proteinase inhibitor synthesis (Davis et al., 1991). The transport of radioactively labeled SYS is blocked by *p*-chloromercuribenzene sulfonic acid (PCMBS), an inhibitor of apoplastic phloem loading in plants (Narvaez-Vasquez et al., 1994; Doares et al., 1995). Therefore, it was proposed that upon wounding, SYS is processed from PS, loaded into the sieve elements and transported to unwounded tissues following source–sink directions (Ryan, 2000). However, grafting experiment involving wound-response tomato mutants and WT plants indicated that the graft-transmissible signal might be JA or a related compound derived from the octadecanoid pathway (Li et al., 2002). Therefore, investigation of the identity of the systemic internal signal in defense responses in tomato remains an interesting problem.

Here, we demonstrated the occurrence of transgenic *PS* mRNA in leaves located at considerable distances from the initial site of its generation in tomato plants using a transient *Agrobacterium*-infiltration assay system. We also showed that the PS-GFP can be processed to release SYS-GFP and Leu178 is essential for PS-GFP processing.

## Materials and Methods

### Plant Materials and Growth Conditions

Tomato (*Lycopersicon esculentum*) and tobacco (*Nicotiana benthamiana*) plants were grown in soil at 21°C with a 16-h light/8-h dark cycle. For the experiments 1-month-old plants were used.

### Construction of Binary Vector

**FIGURE 1:**
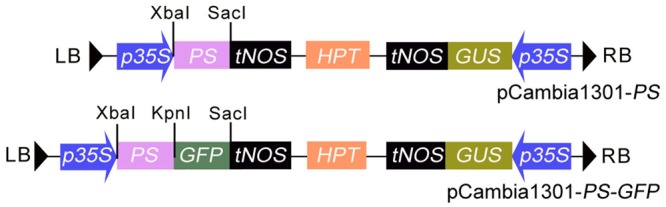
The structure of the T-DNA region in the binary vectors of pCAMBIA1301-*PS* and pCAMBIA1301-*PS-GFP. HPT, Hygromycin phosphotransferase* gene; *GUS, β-glucuronidase* gene; *p35S*, CaMV 35S promoter; *tNOS*, terminator of *nopaline synthase* (*NOS*) gene; LB and RB: the left and right border of the T-DNA.

*PS* cDNA lacking the nucleotide sequence encoding the four C-terminal amino-acids (NNKL) was amplified by PCR from the pGA643 vector containing the *PS* gene using the primers listed in **Table 1**. The PCR product was digested and subcloned as an Xba I/Kpn I fragment into an Xba I/Kpn I-digested pGFP221 vector (Liu et al., 2007), resulting in the plasmid p221-*PS-GFP.* The Hind III/EcoR I-digested fragment containing *35S* promoter, *PS-GFP* and nopaline synthase terminator from p221-*PS-GFP* was inserted into the respective sites of pCambia1301 (with GUS and hygromycin-resistance genes), yielding pCambia1301-*PS-GFP* (*pro35S::PS-GFP*) (**Figure 1**). To generate *pro35S::PS* construct, *PS* cDNA coding region was amplified from pCambia1301-*PS-GFP* and the PCR products cloned into the Xba I and Sac I sites of pCambia1301-*PS-GFP* to substitute the *PS-GFP* fragment (**Figure 1**). The resulting plasmid was transformed into competent *Escherichia coli* strain DH5α and the resulting clones were transformed into *Agrobacterium tumefaciens* strain GV3101 by electroporation.

**Table 1:** List of primers used in this study.

Gene/Terminator	Primer sequence (5′—>3′)	Use	Location (Gene/Terminator/Plasmid)
*PS*	F: tctagaATGGGAACTCCTTCATATGATATCAAAAAC		1..30 *(PS)*
	R: ggtaccCGTCTGTTTGCATTTTGGGAGGATC	Subcloning	564..5S8 *(PS)*
	R: gagctcCGTCTGTTTGCATTTTGGGAGGATC	Subcloning	564..58S *(PS)*
*PS-GFP*	F: gtcgacATGGGAACTCCTTCATATGATATCAAAAAC		1..30 *(PS)*
	R: gcggccgcTTACTTGTACAGCTCGTCCATGC	Subcloning	689…711 *(GFP)*
*SYS-GFP*	F: gtcgacATGGCTGTTCAATCAAAACCTCCAT	Subcloning	535..555 *(PS)*
*Tubulin 8*	F: CGTGGATCACAGCAATACAGAGCC	Amplification	935..958 *(Tubulin 8)*
	R: CCTCCTGCACTTCCACTTGGTCTTC	(RT-PCR)	1427..1451 *(Tubulin 8)*
*PS(L178A)*	R:TCACGCTTTGATGGAGGTTTTGATTGAACAGCAG	Gene	
	CATCTTCTCGTACTATAATTTTCTC	Mutagenesis	507..566 *(PS)*
*PS(L178D/A179E)*	R: TCACGCTTTGATGGAGGTTTTGATTGAACCTCAT	Gene	
	CATCTTCTCGTACTATAATTTTCTC	Mutagenesis	507..566 *(PS)*
*NOST*	R: CGCGCGCGATAATTTATCCTAG	Amplification	210..230 *(NOS terminator)*
*GUS*	F: TGGATCGCGAAAACTGTGGA	Amplification	276..295(pCambia 1301)
	R: TCCAGTTGCAACCACCTGTT	(RT-PCR)	870..889 (pCambia 1301)

*The primers (F, forward primer; R, reverse primer) were designed using Primer 5.0 software. The restriction enzyme sites are underlined.*

### Directed Point Mutation

Plasmid pCambia1301-*PS-GFP* was used as the template for site-directed mutagenesis by PCR. Mutations of Leu178Ala and Leu178AspAla179Glu were introduced into the *PS* sequence by a PCR overlap method. The first-round PCR was performed with the 5′ end primer (PS-forward) and 3′ end primer containing the mutated codon(s) [PS(L178A) or PS(L178DA179E), respectively]. The full length of the modified *PS* fragments were amplified in the second-round PCR using the product of the first-round PCR and the 5′ end primer (PS-forward) and 3′ end primer(PS-reverse), the latter primer partly overlapped with the primers PS(L178A) and PS (L178DA179E). All primers used are listed in **Table 1**. The altered sequence was excised using Xba I and Kpn I. The resulting DNA fragments were then purified and replaced the *PS* sequence of pCambia1301-*PS-GFP*. The resultant plasmids were then transformed into *A. tumefaciens* strain GV3101 by electroporation. All PCR-derived modifications of the *PS* cDNA sequence were verified by DNA sequencing.

### Agro-Infiltration

*A. tumefaciens* GV3101 containing the binary vector was grown overnight in Luria-Bertani (LB) medium with the appropriate antibiotics. The bacteria were briefly spun down (5000 g, 15 min) and re-suspended in suspension buffer (10 mM MES-KOH, pH 5.2, 10 mM MgCl_2_, 100 μM acetosyringone) to an OD_600_ of 0.5 and left for at least 3 h at room temperature. The *Agrobacterium* suspension was infiltrated into lower leaves of tomato or *N. benthamiana via* a needle-less 1-mL syringe. The young developing leaf at the top of each plant was kept non-infiltrated. The infiltrated and non-infiltrated leaves were then harvested separately and total RNA and protein were extracted for RT-PCR and immunoblot analysis.

### Protein Purification

The coding regions of *PS-GFP* and *SYS-GFP* were amplified respectively from the pCambia1301-*PS-GFP* vector by PCR using the primers listed in **Table 1**. The PCR products were digested and subcloned as fragments into a Sal I/Not I-digested pET-28b(+) vector to produce the prokaryotic expression vectors, which were used to transform *E. coli* Rosetta (DE3) cells. Expression of recombinant proteins was induced by 0.1 mM isopropyl β-D-1-thiogalactopyranoside for 16 h at 16°C. Bacteria were collected by centrifugation and lysed *via* ultrasonication before the recombinant proteins were affinity purified using Ni-NTA agarose (QIAGEN, Germany) according to the manufacturer’s manual. The purified proteins contained one 6 × His affinity tag at its N-terminus.

### Immunoblot Analysis

Total protein extracts were obtained by grinding 100 mg leaf tissues in protein extraction buffer [20 mM Tris-HCl, pH 7.5, 5 mM ethylenediaminetetraacetic acid, 5 mM ethylene glycol tetraacetic acid, 10 mM dithiothreitol, 0.05% sodium dodecyl sulfate (SDS), and 1 mM phenylmethylsulfonyl fluoride]. The extracts were centrifuged for 10 min at 4°C, and the resulting supernatants were loaded on SDS-polyacrylamide gel electrophoresis (SDS-PAGE) gels with loading buffer. After electrophoresis, the separated proteins were transferred to a nitrocellulose membrane for 2 h. The membrane was then incubated with 1:4000 anti-GFP antibodies (Sigma Sigma-Aldrich, St. Louis, MO, United States) in phosphate-buffered saline (pH 6.9). Horseradish peroxidase-conjugated secondary antibody (Sigma-Aldrich) was used at 1:5000 dilution and the results were visualized and interpreted using an enhanced chemiluminescence detection system according to the manufacturer’s recommendations (Applygen Technologies Inc., Beijing, China). The intensity of bands on the blots was quantified by densitometry of images using ImageJ software (Media Cybernetics, San Diego, CA, United States).

### Confocal Laser Scanning Microscopy

GFP signals in leaves were visualized using a TCS SP5 confocal laser-scanning microscope (Leica, Oberkochen, Germany). The excitation wavelength for GFP was 488 nm and emission wavelength was collected between 500 and 530 nm. Images were edited using the LAS AF Lite image browser (Leica) and Adobe Photoshop CS3 (Adobe Systems, San Jose, CA, United States).

## Results and Discussion

### The *PS* Messenger RNA Is Mobile in Tomato

**FIGURE 2:**
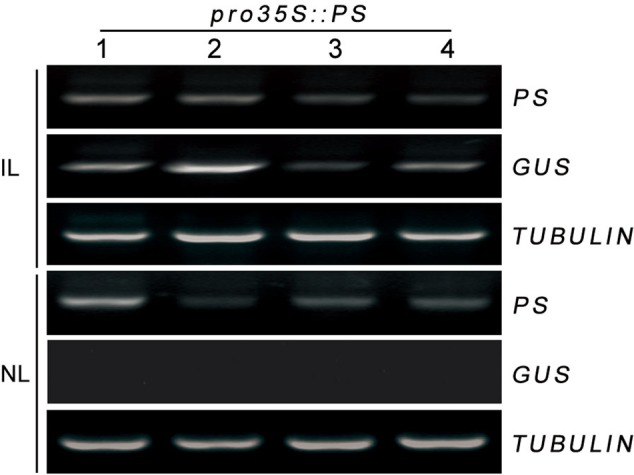
RT-PCR analysis of mRNA levels of *PS* in infiltrated and non-infiltrated leaves of tomato. In a 1-month-old plant, lower leaves were infiltrated with *Agrobacteria* harboring *pro35S::PS* plasmid. The infiltrated and non-infiltrated leaves were harvested at 4 days post-infiltration and the transcript levels of *PS* were analyzed by RT-PCR, respectively. Expression of *TUBULIN* and *GUS* served as controls. IL, infiltrated leaves; NL, non-infiltrated leaves.

Trafficked mRNA molecules in plants can act as long-distance signals in the control of developmental processes and in responses to environmental cues (Kehr and Buhtz, 2008). *Prosystemin* (*PS*) mRNA was exclusively present in the vascular bundle in wounded or methyl jasmonate-treated leaves of tomato (Jacinto et al., 1997). We hypothesized that the localization of *PS* mRNA may help its load into the phloem, where it could be readily transported throughout the plant. A transient *Agrobacterium*-infiltration assay system was used to investigate this hypothesis in tomato. We monitored whether *PS* mRNA produced transiently in infiltrated leaves can move to non-infiltrated leaves in tomato. Lower leaves of tomato were infiltrated with a culture of *A. tumefaciens* carrying *pro35S::PS* construct and the infiltrated and the non-infiltrated leaves (young developing leaf at the top of each plant) were collected separately at 4 days postinfiltration for RT-PCR analysis. To discriminate the *pro35S::PS* transgene from endogenous *PS* mRNA, specific primers against *PS* and non-plant nopaline synthase (*NOS*) terminator (Banerjee et al., 2006; Lu et al., 2012) were used for PCR reactions. We found that the infiltrated leaves accumulated high levels of transgenic *PS* mRNA and the non-infiltrated leaves showed detectable transgenic *PS* mRNA (**Figure 2**). To exclude the possibility that the detectable *PS* mRNA in the non-infiltrated leaves is due to contamination during sample infiltration and collection, we tested the transcriptional levels of β-glucuronidase (GUS) reporter gene harbored by vector backbone pCambia1301 of *pro35S::PS* construct (see Materials and Methods). As expected, *GUS* mRNA was detected only in the infiltrated leaves, but not in the non-infiltrated leaves (**Figure 2**). These results suggested that transgenic *PS* mRNA can be transported over long distance in tomato.

**FIGURE 3:**
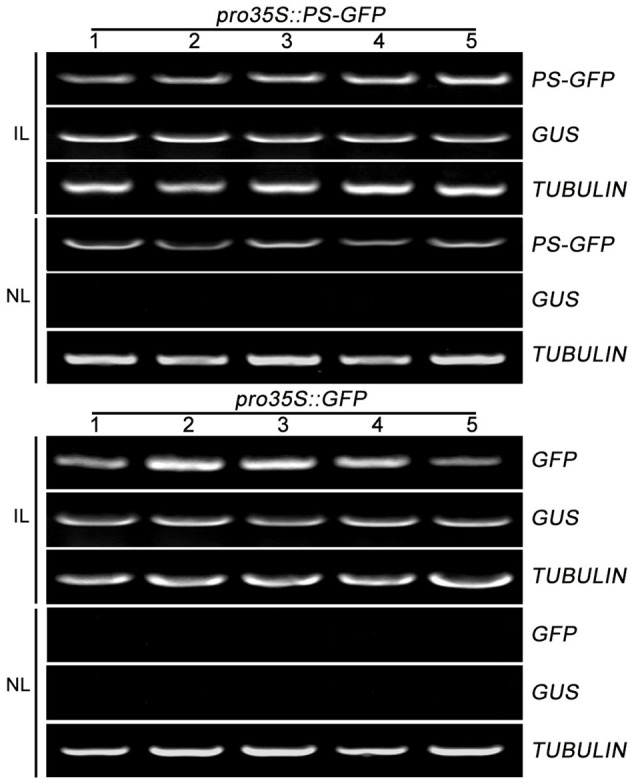
The mobility of *PS-GFP* mRNA in tomato. RT-PCR analysis of mRNA levels of *PS-GFP* and *GFP* in infiltrated and non-infiltrated leaves of tomato. Expression of *TUBULIN* and *GUS* served as controls. IL, infiltrated leaves; NL, non-infiltrated leaves.

To further confirm the movement feature of transgenic *PS* mRNA, we tested the mobility of *PS* mRNA fused with *GFP* mRNA which was known to be immobile within plants (Huang and Yu, 2009). We generated a construct (*pro35S::PS-GFP*) encoding a PS-GFP fusion in which the four C-terminal amino acids (NNKL) excluded in/out SYS sequence, of PS were replaced by GFP under the control of the constitutive promoter *35S*. Construct *pro35S::GFP* encoding free *GFP* under the control of the constitutive promoter *35S* were used as a negative control. We found that the infiltrated leaves accumulated high levels of *PS-GFP* mRNA and the non-infiltrated leaves showed detectable *PS-GFP* mRNA (**Figure 3**). However, when *pro35S::GFP* plasmid was applied, free *GFP* transcripts were detected in all infiltrated leaves but not detected in non-infiltrated leaves (**Figure 3**). These results suggested the movement of *PS-GFP* mRNA is correlated with the *PS* sequence but not with the *GFP* sequence.

### Expression of GFP Fusion Protein in Leaves of *N. benthamiana*

**FIGURE 4:**
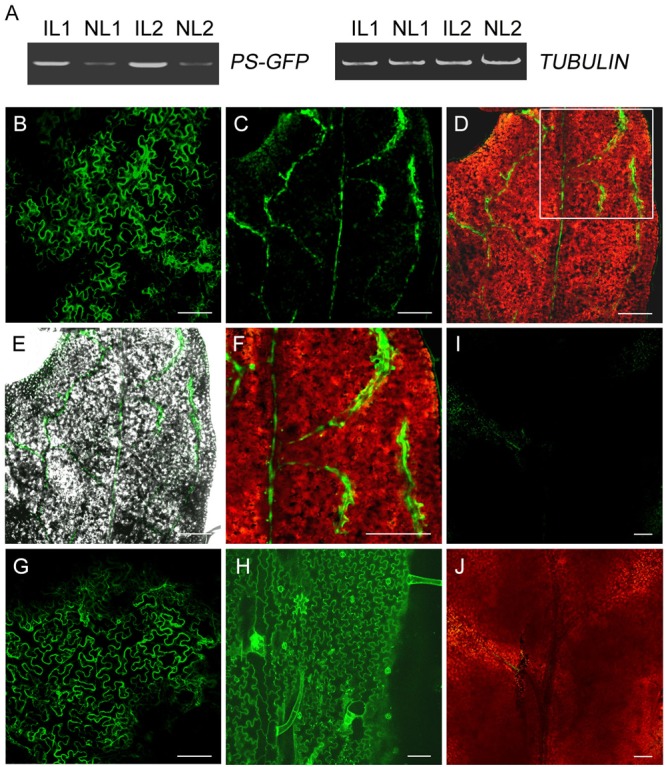
Expression of GFP fusion protein in leaves of *N. benthamiana*. **(A)** RT-PCR analysis of levels of *PS-GFP* transcripts in infiltrated and non-infiltrated leaves of *N. benthamiana.* IL, infiltrated leaves; NL, non-infiltrated leaves. **(B,G)** Fluorescence signals in leaves infiltrated with *Agrobacteria* carrying *pro35S::PS-GFP* **(B)** or *pro35S::GFP* **(G)** construct. **(C–F)** Confocal microscopy demonstrated that PS-/SYS-GFP protein was primarily detectable the veins of non-infiltrated developing leaf of *N. benthamiana* whose lower leaves were infiltrated with *Agrobacteria* harboring *pro35S::PS-GFP* plasmid. Merged image of GFP fusion protein (green) and chloroplast autofluorescence (red) or the corresponding bright-field image is shown in **D** and **E**, respectively. Enlarged view of the veins of non-infiltrated leaf of the inset in **D** is shown in **F**. **(H)** Fluorescence signals in non-infiltrated leaf of *N. benthamiana* whose lower leaves were infiltrated with *Agrobacteria* harboring *pro35S::GFP* plasmid. Note that free GFP fluorescence was distributed evenly on the cell surface of leaf pavement cells. **(I,J)** Control image of non-transgenic leaf of *N. benthamiana* taken at the same laser intensity and exposure time as that in **B–H**. Scale bars: 100 μm in **B** and **G–J**, 200 μm in **C–F**.

To investigate whether PS-GFP fusion protein is present in the developing non-infiltrated leaves when *PS-GFP* mRNA is produced in the infiltrated leaves, we observed PS-GFP fluorescence in the non-infiltrated tomato leaves by confocal microscope. Unexpectedly, tomato leaf cells exhibits strong autofluorescence either before or after agro-infiltration (Zizkova et al., 2015). We then performed agro-infiltration of *pro35S::PS-GFP* culture using tobacco *N. benthamiana* plants. Like in tomato, we detected transgenic *PS* mRNA in the non-infiltrated new leaves freshly emerged from the top axial bud of tobacco plants (**Figure 4A**). Confocal microscopic observation showed that the fluorescence of PS-GFP was found in most cells of the infiltrated leaf epidermis (**Figure 4B)**. However, PS-GFP fluorescence was present exclusively in the veins of the developing non-infiltrated leaves (**Figures 4C–F**). In contrast, when *pro35S::GFP* plasmid was applied, free GFP fluorescence was found in all the cells of both the infiltrated and the non-infiltrated developing leaves (**Figures 4G–J**), supporting the previous conclusion that free GFP protein can move over long distance in plants (Imlau et al., 1999). Although we do not provide conclusive evidence for PS protein trafficking because it is not easy to characterize movement of a protein if its corresponding mRNA is mobile, PS-GFP in the non-infiltrated leaves is probably required for the systemic SYS signaling.

### PS-GFP Protein Can Be Processed in Tomato

**FIGURE 5:**
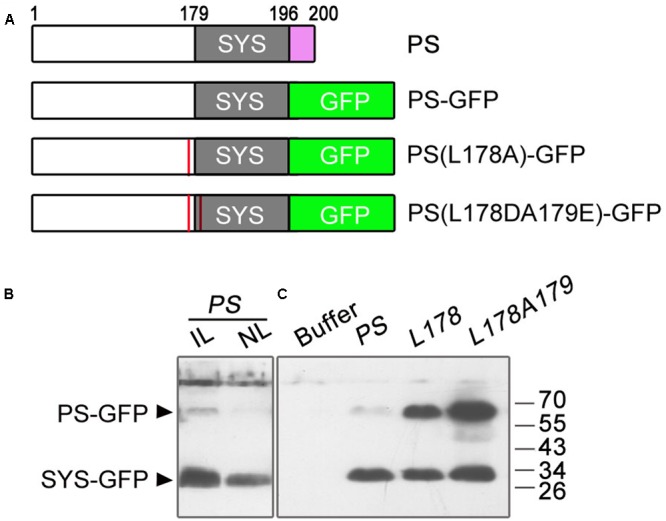
Immunoblot analysis of GFP fusion proteins in infiltrated and non-infiltrated leaves of tomato. **(A)** Summary of the *PS* constructs tested for immunoblot analysis of GFP fusion proteins. The wild-type PS consists of 200 amino acids. The numbers above the boxes denote the amino acid number in the wild-type sequence. The gray boxes represent the location of SYS peptide in the various PS proteins. Substitution of the last four amino acids (NNKL, purple box) of the PS protein with the GFP sequence (green box) was performed to produce the PS-GFP fusion protein. The red lines in PS(L178A)-GFP and PS(L178DA179E)-GFP denote replacement of Ala for Leu178 proximate to the N-terminus of the SYS domain and substitution for Leu178 and Ala179 (the first residue of the SYS domain) by Asp and Glu in PS protein, respectively. **(B)** Immunoblots with GFP antibodies in leaves infiltrated and non-infiltrated leaves of tomato. In a 1-month-old plant, lower leaves were infiltrated with *Agrobacteria* harboring *pro35S::PS-GFP (PS)* plasmid. The infiltrated and non-infiltrated leaves were harvested at 4 days post-infiltration and the protein levels of *PS* were analyzed by Immunoblot, respectively. IL, infiltrated leaves; NL, non-infiltrated leaves. **(C)** Immunoblots with GFP antibodies in leaves infiltrated with suspension buffer (see Materials and Methods), *pro35S::PS-GFP* (*PS*) or *PS* mutant plasmids [*L178, pro35S::PS(L178A)-GFP*; *L178A179, pro35S::PS(L178DA179E)-GFP*]. The bands of PS-GFP and SYS-GFP are indicated.

To test whether the deletion of the amino acids NNKL has an effect on the cleavage of PS-GFP into SYS-GFP, we performed immunoblot analysis using GFP antibodies on the tomato leaves infiltrated with *A. tumefaciens* harboring the *pro35S::PS-GFP* plasmid and on the non-infiltrated leaves. Previous studies showed that PS protein produced in *E. coli* or expressed in tobacco was detected as a ∼40 kDa protein (much larger than its predicted size of 23 kDa) (Delano et al., 1999; Rocco et al., 2008). In this study, we detected one weak band at the expected molecular weight of full length PS-GFP (about 67 kDa based on the abnormal migration of PS, 40 kDa for PS plus 27 kDa for GFP) and one strong smaller band at ∼30 kDa in both infiltrated and non-infiltrated leaves (**Figures 5A,B**). In view of migration of the fusion proteins PS-GFP and SYS-GFP produced in *E. coli* as ∼67 kDa and ∼30 kDa polypeptides with SDS-PAGE, respectively (**Supplementary Figure S1**), we presumed that smaller protein detected in the agro-infiltrated leaves is the processed form (SYS-GFP) of PS-GFP protein, and its retarded mobility is probably due to the highly hydrophilic nature of the SYS peptide.

### LEU178 is Involved in PS Processing

To confirm whether the protein detected in the *pro35S::PS-GFP* infiltrated tomato leaves was indeed SYS-GFP, we disrupted the putative processing site(s) of PS by mutating the amino acids bordering SYS peptide. The presumed junction (Leu178-Ala179) between the precursor and the processed peptide likely contains a recognition site for cleavage, as found for the plant peptide hormone RALF23 (Srivastava et al., 2009). Therefore mutagenesis of PS cDNA was performed at sequences encoding the Leu178-Ala179 junction (**Figure 5A**). We constructed two mutant plasmids, one containing a replacement of Ala for Leu178 proximate to the N-terminus of the SYS domain, and the other in which Leu178 and Ala179 (the first residue of the SYS domain) were replaced by Asp and Glu, respectively (**Figure 5A**). Immunoblot analysis showed that as compared with leaves expressing *pro35S::PS-GFP* both simultaneous substitution of Leu178 and Ala179 and single substitution of Leu178 resulted in enhanced band of ∼67 kD (**Figure 5C**), suggesting that Leu178 is involved in PS processing in tomato.

Our evidence shows that transgenic *PS* mRNA can be mobile within the plants, and more importantly, the mobile transgenic *PS* mRNA may undergo translation into protein, suggesting that translocated *PS* mRNA from the stress-exposed tissues probably function as a long-distance signal to prime systemic defense resistance in tomato. However, we should note that in addition to *PS* mRNA, other molecules operating independently of or in parallel with the SYS signaling pathway might also travel throughout the tomato plant suffering from wounding. Previous work demonstrated that JA can travel throughout the plant and induce systemic wound responses in tomato (Li et al., 2002) although JA biosynthesis or downstream signaling is not essential for systemic acquired resistance in Arabidopsis (Attaran et al., 2009). Phloem-loaded-radiolabeled SYS was distributed throughout the plants within a few hours within the plant (Narváez-Vásquez et al., 1995). In addition, evidence has shown that volatile organic compounds play a role in within- and between-plant signaling (Heil and Ton, 2008). Taken together, it seems evident that within-plant signaling by *PS* mRNA can synergize the response to other signaling molecules such as JA or SYS peptide to regulate systemically expressed resistance.

## Author Contributions

All authors listed, have made substantial, direct and intellectual contribution to the work, and approved it for publication.

## Conflict of Interest Statement

The authors declare that the research was conducted in the absence of any commercial or financial relationships that could be construed as a potential conflict of interest.

## Supplementary Material

FIGURE S1 Coomassie-stained SDS-PAGE gel showing the positions of purified PS-GFP and SYS-GFP. Fusion proteins were purified by Ni-chelating affinity chromatography. Both PS-GFP and SYS-GFP expressed in *E. coli* exist in the supernatant (Su) of the lysate and the sediment (Se). Arrowheads indicate the bands of PS-GFP and SYS-GFP with a molecular weight of ∼67 kD and ∼30 kD, respectively. Cr denotes the crude supernatant of *E. coli*. The positions of molecular weight markers are indicated.
