# Supplementary figures and images for: Long-Distance Transport of Prosystemin Messenger RNA in Tomato
Source: Front Plant Sci. 2017 Nov 6;8:1894. doi: 10.3389/fpls.2017.01894 (PMC5681517; doi:10.3389/fpls.2017.01894)

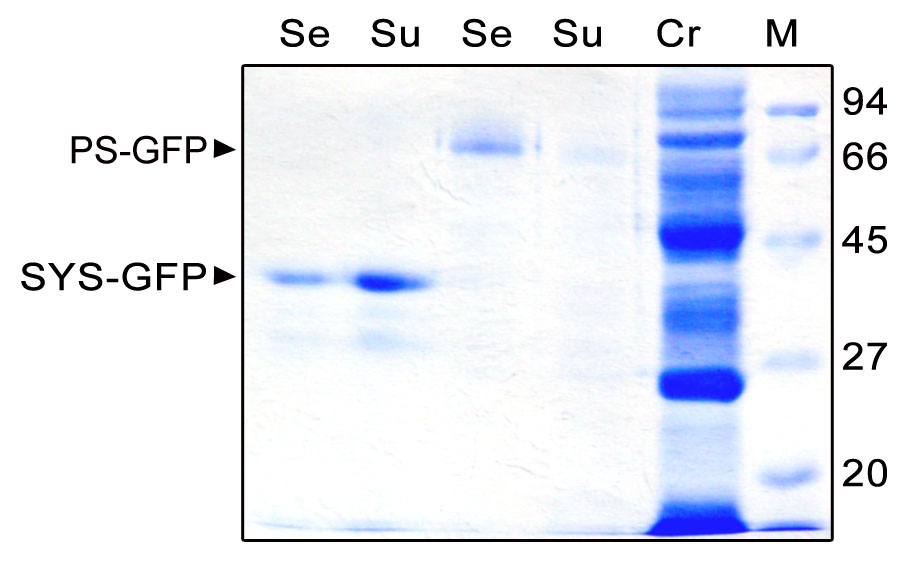

Supplement: FIGURE S1 — Coomassie-stained SDS-PAGE gel showing the positions of purified PS-GFP and SYS-GFP. Fusion proteins were purified by Ni-chelating affinity chromatography. Both PS-GFP and SYS-GFP expressed in E. coli exist in the supernatant (Su) of the lysate and the sediment (Se). Arrowheads indicate the bands of PS-GFP and SYS-GFP with a molecular weight of ∼67 kD and ∼30 kD, respectively. Cr denotes the crude supernatant of E. coli. The positions of molecular weight markers are indicated. [file Image_1.JPEG]
